# Laparoscopic repeat liver resection

**DOI:** 10.1002/ags3.12363

**Published:** 2020-06-10

**Authors:** Zenichi Morise

**Affiliations:** ^1^ Department of Surgery Fujita Health University School of Medicine Okazaki Medical Center Aichi Japan

**Keywords:** hepatocellular carcinoma, laparoscopic liver resection, metastasis, repeat surgery

## Abstract

Recurrence of liver cancers inside the liver are often treated with liver resection (LR). However, increased risks of complications and conversion during operation were reported in laparoscopic repeat LR (LRLR). The indication is still controversial. One multi‐institutional propensity score matching analysis of LRLR vs open repeat LR for hepatocellular carcinoma, two propensity score matching analyses for colorectal metastases, and two meta‐analyses including hepatocellular carcinoma, intrahepatic cholangiocarcinoma, metastases, and other tumors have been reported to date. LRLR was reported with better to comparable short‐term and similar long‐term outcomes. Furthermore, the shorter operation time and the smaller amount of intraoperative bleeding for LRLR was reported for the patients who had undergone laparoscopic rather than open LR as an earlier procedure. The speculations are presented, that complete dissection of adhesion can be dodged and laparoscopic minor repeated LR can minimize the liver functional deterioration in cirrhotic patients. LRLR, as a powerful local therapy, could contribute to the long‐term outcomes of those with deteriorated liver function. However, the procedure is now in its developing stage worldwide and further accumulation of experiences and evaluation are needed.

## Graphical abstract

One multi‐institutional propensity score matching analysis of LRLR vs open repeat LR for hepatocellular carcinoma, two propensity score matching analyses for colorectal metastases, and two meta‐analyses including hepatocellular carcinoma, intrahepatic cholangiocarcinoma, metastases, and other tumors have been reported to date. The speculations are presented that complete dissection of adhesion can be dodged and laparoscopic minor repeated LR can minimized liver functional deterioration in cirrhotic patients. LRLR, as a powerful local therapy, could contribute the long‐term outcomes of those with deteriorated liver function. However, the procedure is now in its developing stage worldwide and further accumulation of experiences and evaluation are needed.

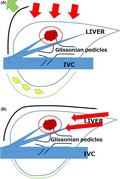

## 1 INTRODUCTION

The neoplastic condition of the chronically injured liver can develop multifocal and metachronous hepatocellular carcinoma (HCC), and metastases of malignancies can also be repeatedly found. Repeated liver resection (LR) is often performed for those conditions without other uncontrollable lesions.^1^, ^2^, ^3^, ^4^

Recently, after the accumulation of experiences and technical/instrumental developments, laparoscopic LR (LLR) was employed more often.^5^, ^6^, ^7^, ^8^ However, there have still been several obstacles in LLR. Surgeons should handle the bulky and weighty liver, fixed inside the limited subphrenic space (rib cage), and invisible tumors and vessels inside it to overcome complicated laparoscopic conditions, such as constrained manipulation, poor tactile sensation, and disorientation under the limited view for whole surgical field.^9^, ^10^ In addition, increased operation time and incidence of bowel injury were reported in surgery with adhesions.^11^, ^12^ Increased morbidity and conversion in re‐do laparoscopic procedures had also been known.^12^, ^13^ Although many laparoscopic re‐do surgeries^12^, ^13^, ^14^, ^15^, ^16^ occurred within indications after technical and instrumental improvements, the indication of laparoscopic repeat LR (LRLR) is still under discussion. Adhesion can disrupt the liver mobilization and the dissections of vessels and Glissonian pedicles (including hepatoduodenal ligament) in LR. Scars and adhesions with the deformity of the liver and structures make the identification of lesions and the important structures problematic. Easy bleeding from the liver capsule causes a suboptimal surgical field during the dissection of adhesion.^17^ Former surgical histories can cause these changes which increase the risks of complications and conversions during LRLR.

## 2 FEATURES OF LLR

**Figure 1:**
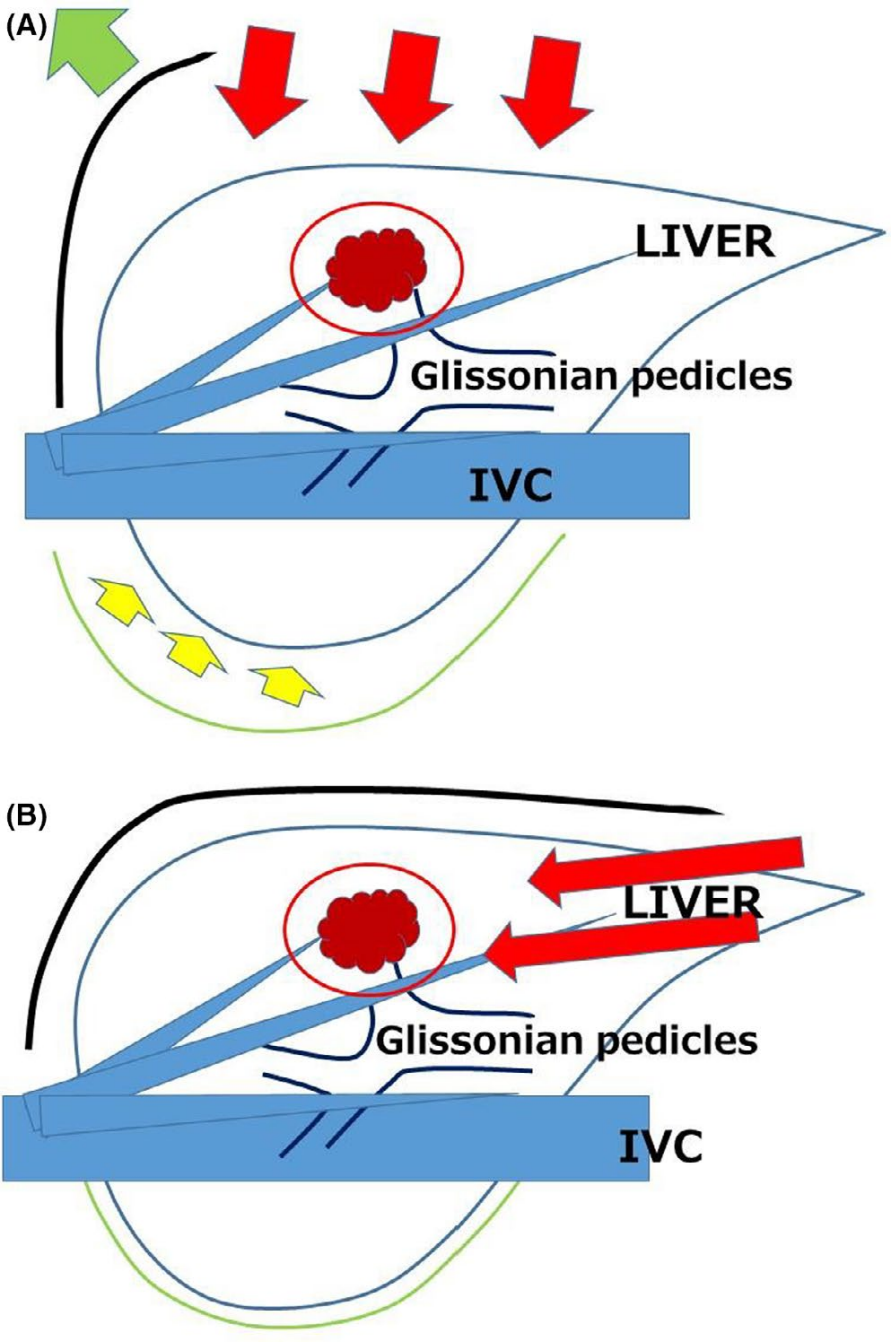
Schema of open liver resection (A) and laparoscopic liver resection (caudal approach, B). Red arrows indicate the directions of view and manipulation in each approach. A. In the open approach, the subphrenic rib cage containing the liver is opened with a large subcostal incision and instruments are used to lift the costal arch up, and the liver is dissected and mobilized (lifted) from the retroperitoneum; B, In laparoscopic caudal approach, the laparoscope and forceps intrude into the subphrenic rib cage from the caudal direction, and the surgery is performed with minimal alteration and destruction of the associated structures. In the same context, direct approach to the tumor in LRLR after minimal adhesiolysis for the surgical space can be facilitated especially in small LRLR. (Modification from Morise Z, Wakabayashi G. First quarter century of laparoscopic liver resection. World J Gastroenterol. 2017;23(20):3581‐3588.^8^)

LLR is reported to be beneficial for patients with chronically injured liver.^18^, ^19^, ^20^ It can minimize the damages to collateral vessels as well as liver parenchyma through its minimal laparotomy, mobilization, and compression. Postoperative ascites and liver failure are reported to be reduced.^21^ LR is performed for the liver, which is protected inside the subphrenic rib cage. The cage is opened by the large abdominal wall incision and the liver is mobilized and moved out in open surgery. However, LLR is performed with the direct intrusion of instruments to the space from caudal direction (Figure 1, “Caudal approach”^22^, ^23^, ^24^). Small targeted area is manipulated with minimum damages to surrounding structures. Postural changes in LLR, under the maintenance of the same view by the adjustment of the laparoscope, can facilitate handling structures using gravity. It also reduces the damage from the compression on liver parenchyma. We set the novel LLR concept of “Caudal approach” and reported posterior sectionectomy in left lateral position.^22^ Our preceding transection of liver parenchyma before mobilization in the position makes exposure of the cutting plane easier. Well‐opened clear view of the transection plane from caudal direction is obtained between the retroperitoneal‐fixed resected liver and the residual liver falling down by gravity. Minimum compression damages and destruction of surrounding structures can be obtained in our approach. The key for those LLR advantages is minimal dissection under the direct access to the small targeted area with postural change.

In a similar framework, direct approach for working space in LRLR, after minimal dissection of adhesion, can be enabled especially in small LRLR.^25^, ^26^, ^27^ Several reports have shown that time and blood loss in LLR were similar in primary and repeat settings,^27^, ^28^ although there are usually large differences between primary and repeat open procedures. Furthermore, the shorter operation time and the smaller intraoperative bleeding for LRLR was reported, when it was applied to the patients who had previously undergone LLR rather than open LR.^29^

## 3 STUDIES OF LRLR AND OUR PROPENSITY SCORE MATCHING ANALYSIS FOR HCC PATIENTS

There is no randomized control trial for LRLR vs open repeat LR (ORLR), although the number of reports is increasing. Our multi‐institutional propensity score matching analysis of LRLR vs ORLR for HCC,^30^ two propensity score matching analyses for colorectal metastases,^31^, ^32^ and two meta‐analyses that included cases of HCC, intrahepatic cholangiocarcinoma, metastases, and other tumors^33^, ^34^ have been published to date (Table 1). These studies showed that LRLR reduced bleeding, had less or similar morbidity, and shortened or similar length of stay with the equivalent long‐term outcomes.

**Table 1:** Summary of previous reports of LRLR (propensity score matching analyses & meta‐analyses)

Author	year	Journal	Study design	Disease	Number (ORLR: LRLR)	Short‐term outcomes	Long‐term outcoms
Morise Z	2020	Br J Surg	Multicenter PSM	HCC	934:648	blood loss: LRLR favor operation time: ORLR favor	OS no significant difference DFS no significant difference
Peng Y	2019	Medicine	Meta‐analysis	HCC, CRLM, others	396:364	blood loss, BT, hospital stay, morbidity: LRLR favor	OS: not available DFS: comparable
Peng L	2019	Surg Oncol	Meta‐analysis	HCC, CRLM, others	271:172	blood loss, BT, hospital stay: LRLR favor operation time: ORLR favor	OS and DFS: not available (comparable in systematic review)
von der Poel MJ	2019	Br J Surg	Multicenter PSM	CRLM	154:271	blood loss, operation time, hospital stay: LRLR favor	OS and DFS: not available
Hallet J	2017	World J Surg	Multicenter PSM	CRLM	349:27	Comparable (operation time, BT, morbidity)	OS: not available DFS: comparable

Abbreviations: BT, blood transfusion rate; CRLM, colorectal carcinoma liver metastasis; DFS, disease‐free survival; HCC, hepatocellular carcinoma; LRLR, laparoscopic repeat liver resection; ORLR, open repeat liver resection; OS, overall survival; PSM, propensity score matching analysis.

Journal names: Br J Surg, British journal of surgery; Surg Oncol, Surgical oncology; World J Surg, World journal of surgery

The magnified view and strained adhesion by pneumoperitoneum in LLR can facilitate meticulous dissection^35^ and also laparoscopic approach can make complete dissection of adhesion unnecessary as mentioned above.^24^, ^25^ Contrary to the LR for metastases, minor LR for the fibrotic liver with poor functional reserve and collaterals is frequently applied for HCC. The advantages of LLR are reported especially for the HCC patients’ management during the long history with repeat oncogenesis.^35^, ^36^

We conducted the first multi‐institutional propensity score matching analysis of LRLR vs ORLR for HCC^30^ with 1582 repeat LR cases at 42 high‐volume centers around the world. It showed that LRLR was not inferior to ORLR in short‐ and long‐term outcomes and LRLR is feasible for selected patients. The analysis was performed on an intention‐to‐treat basis, and the conversion rate was 3.8%. This low conversion rate may derive from the selection. This study showed that LRLR was generally applied to patients of poor performance status with poor liver function but with favorable factors related to tumors and surgical procedures. Our analysis also revealed notable differences between centers in the number and percentage of LRLRs, even without the correlation between numbers and percentages. The indications for LRLR in patients with recurrent HCC varied between centers, even though all are high‐volume centers. Western and Eastern centers registered 96 and 552 LRLR patients, respectively. Western patients with smaller number of tumors and better liver function were applied more extensive resections and had significantly better survival time. The whole patients after matching (LRLR plus ORLR), in comparison to the whole patients before matching, were shown to have had better performance status, liver function, and factors related to tumors and surgical procedures. Surgeons exercise discretion based on characteristics of the particular case and the experience of their own centers. The patients after matching might have been favorable patients that would have made them eligible for either LRLR or ORLR depending on the experience of each center. The overall survival curve of LRLR group after matching was clearly separated and better than that of ORLR group after matching, even though the difference was not significant (median 12.55 vs 8.94 years; *P* = .086), though the disease‐free survivals were similar (1.79 vs 2.32 years; *P* = .517). LRLR patients after matching have better liver function, though LRLR patients before matching were selected with poorer liver function and have comparable overall survival to ORLR patients before matching (10.04 vs 8.94 years; *P* = .297). LRLR patients after matching might have been able to undergo repeated treatments due to less adhesion and less liver‐functional deterioration possibly due to laparoscopic approach. Our study with propensity score matching showed that LRLR results in less blood loss, a longer operation time, and similar long‐term outcomes. With the exception of morbidity and hospital stay, our data were comparable to previous reports. Decreased morbidity is considered as one of the advantages of LLR for HCC patients. However, our patients after matching have a favorable liver function, and thus, the influence of LLR on morbidity might be lower. Also, the differences in hospital stay between centers and/or areas, possibly due to insurance systems and hospitalization practices, were large. This might be the reason why there is no difference in hospital stay.

The number and percentage of LRLRs for HCC differed greatly between centers in our study. The number of LRLRs per center ranged from 0 to 67 (median 10). LRLR accounted for 41.0% of all repeat LR cases and from 0% to 100% (median 57.1%) of the cases undertaken at each center. Also, no correlation was found between the number and percentage of LRLRs performed at each center (*P* = .349). This is perhaps because that indications differ depending on each center's experiences and because patient populations differ in terms of the prevalence of HCC. LRLR for HCC patients is now being applied only for the patients with favorable conditions depending on each center's experiences. Therefore, we believe that this procedure is still in its developing stage worldwide. Among our own experience of 33 repeat and 12 three‐or‐more‐time repeat LLR cases, there were three cases with anatomical resection or resections exposing major vessels after previous anatomical resection who developed bile leakage and >30 days hospital stay. Anatomical alterations on major vessels with scars and adhesions may have big influences on later resections also exposing them. Evaluations of such setting of LRLR should be required after the accumulation of more experiences in this area.

Nevertheless, our international multicenter propensity score analysis showed that neither short‐ nor long‐term outcomes of LRLR are inferior to those of ORLR. A large‐scale study conducted after further establishment of the procedure and greater accumulation of experience is needed to confirm the role of LRLR.

## DISCLOSURE

Conflict of Interest: The author declares no conflicts of interest related to this publication.

Author Contributions: Morise Z. collected the data and wrote this paper.
